# Image fusion of IR and optical microscopy for mapping of biomolecules in tissue

**DOI:** 10.1039/d1an01161h

**Published:** 2021-08-31

**Authors:** Safaa Al Jedani, Conor A. Whitley, Barnaby G. Ellis, Asterios Triantafyllou, Caroline I. Smith, Philip J. Gunning, Peter Gardner, Janet M. Risk, Peter Weightman, Steve D. Barrett

**Affiliations:** Department of Physics, University of Liverpool L69 7ZE UK S.D.Barrett@liverpool.ac.uk; Department of Pathology, Liverpool Clinical Laboratories, University of Liverpool Liverpool L69 3GA UK; Department of Molecular and Clinical Cancer Medicine, Institute of Systems, Molecular and Integrative Biology, University of Liverpool L3 9TA UK; Manchester Institute of Biotechnology, University of Manchester 131 Princess Street Manchester M1 7DN UK

## Abstract

It is shown that a pixel-level image fusion technique can produce images that combine the spatial resolution of optical microscopy images of haematoxylin and eosin (H&E) stained tissue with the chemical information in Fourier transform infrared (FTIR) images. The fused images show minimal distortion and the higher spatial resolution of the H&E images overcomes the diffraction limit on the spatial resolution of the FTIR images. A consideration of the FTIR spectra of nucleic acids and collagen can explain the changes in contrast between non-cancerous oral epithelium and underlying stroma within fused images formed by combining an H&E stain of oral tissue with FTIR images of the tissue obtained at a number of wavenumbers.

## Abstract 2

We demonstrate image fusion of H&E stains (left) with FTIR images (centre) to create fused images (right) that have both the spatial resolution of the H&E and the chemical information of the FTIR.

## Graphical abstract

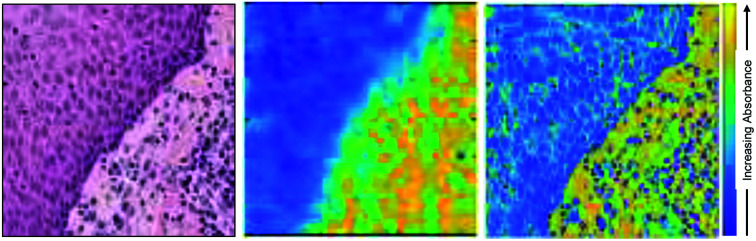

## Introduction

A standard approach for examining excised human tissues is to stain sections with haematoxylin and eosin (H&E) and examine them with optical light microscopy. This approach results in the preservation of morphology and spatial relationships of cells and tissues. The H&E stain highlights the nucleic acid and protein content of the tissues at blue and red visible wavelengths, respectively. H&E staining, together with other histochemical, immunohistochemical and molecular techniques are the “gold standard” for the histopathological diagnosis of cancer and have provided a vast wealth of information on the chemistry of tissues. Expanding the wavelength range of tissue imaging would also convey additional information and there has been progress in the application of infrared (IR) techniques to the examination of tissue in order to exploit the association of particular IR wavelengths with specific chemical moieties. Fourier transform infrared (FTIR) spectroscopy has considerable potential in this regard^1–6^ but, since IR wavelengths are longer than visible wavelengths, the diffraction-limited spatial resolution that can be obtained is far worse than for light microscopy. This limit can be overcome by near-field techniques, but these are time consuming and limited to small areas.^6–8^ Although the registration of images without combining the information within them^9–12^ can assist interpretation, this does not provide full integration of image modality and hence this limits the application to merely annotation of the IR images.

Previous work has focused on developing instruments that combine multi-modality such as correlative microscopy^13,14^ and positron emission tomography with magnetic resonance imaging.^15^ Recent developments have included combining IR with visible light in order to probe the IR-induced thermal expansion at a spatial resolution exceeding that of conventional IR microscopy. However, this technique has the risk of overheating and damaging sensitive samples.^16,17^

Full integration can be achieved using image fusion, which involves merging geometrically registered multi-modal images into a single image that incorporates all of the important information from multiple images. Pixel-level fusion combines the information pixel-by-pixel from different image data sources into new synthetic data^18,19^ and has been used to improve clinical diagnostics by merging computed tomography and single-photon emission computed tomography.^20^ Also, the sharpening of mass spectrometry images using fusion produces results comparable to those obtained from high-resolution ion distribution images, but at lower cost.^21–23^

A range of image fusion methods have been developed, broadly divided into two groups; multiscale-decomposition (MSD) and non-multiscale-decomposition (NMSD). The key distinction between these two approaches lies with how information is extracted from both input images and then merged into the fused image. MSD combines the input images’ multiscale decompositions and then constructs a composite multiscale representation from these images; the fused image is acquired by taking an inverse multiscale transform. Examples of MSD methods for image fusion include pyramid transformation, discrete wavelet transformation and curvelet transformation. However, there are reports that MSD might result in spatial distortions.^18,19,24^

Other methods which are not dependent on the multiscale transformations are considered as NMSD. Most of these methods rely on statistical approaches, such as estimation theory-based methods, linear methods (*e.g.*, principal component analysis and regression), nonlinear methods and artificial neural networks. An element of NMSD methods is known as component substitution; these elements depend on substituting the intensity components from the low-resolution image with those from the high-resolution image. However, these elements suffer from ‘colour distortion’ that affects the representation of the fusion results.^18,19^ In this study, we apply image fusion to merge images through linear regression models. The advantages of regression methods are not limited to combining complementary imaging modalities in order to enhance spatial resolution; they can also be used as machine-learning models that can predict one modality from another.^21,25^

In the present investigation, we show that pixel-level full image integration, or fusion, can generate images which combine the spatial resolution of optical images of H&E stained tissue with the chemical information in FTIR images, providing an insight into the chemical structure of tissues. Fusing IR and optical images could therefore open IR microscopy data up to wider applications using these methods.

## Materials and methods

### Sample preparation and data acquisition

Archival, formalin-fixed, paraffin-embedded (FFPE) tissues were obtained from the Pathology Laboratory at the Royal Liverpool and Broadgreen Hospitals University Trust, following informed consent and under ethical approval (REC number EC 47.01).

Three cases with oral squamous cell carcinoma (OSCC) were selected for analysis:

(A) hyperplastic non-tumoural epithelium located adjacent to OSCC;

(B) cervical lymph node metastasis of OSCC;

(C) primary OSCC infiltrating stroma.

Regions of interest (ROIs) were identified by light microscopy on sections routinely prepared and stained with H&E. Cores of 1 mm diameter corresponding to the ROIs were then obtained from the FFPE blocks using a Beecher MTA-1 (Beecher Instruments Inc., Sun Prairie, WI, USA) tissue microarrayer for constructing a tissue microarray block.

Sequential 5 μm sections of the FFPEs were cut with a microtome from the tissue microarray block and mounted onto either calcium fluoride (CaF_2_) disks for IR imaging or onto charged glass slides for H&E staining. While sections for H&E were deparaffinisated, sections for FTIR remained in paraffin wax to minimise further changes in hydration and structure of the samples. Four serial sections were utilised and comprised two sections for FTIR imaging sandwiched between two sections that were stained with H&E and scanned using an Aperio CS2scanner (Leica Biosystems, Milton Keynes, UK). Using sequential slices helps minimise any differences between the ROIs in the two imaging modalities.

IR imaging was carried out at room temperature and <1% humidity using an Agilent Cary 670-FTIR spectrometer in conjunction with an Agilent Cary 620-FTIR imaging microscope (Agilent, Stockport, UK) with a LN_2_-cooled 128 × 128-pixel mercury-cadmium-telluride (MCT) focal plane array that generates images with a pixel size of 5.5 μm. Images were acquired at a spectral resolution of 6 cm^−1^ over a spectral range of 3800–990 cm^−1^ using 128 co-additions per image. The output of the imaging spectrometer is a data cube comprising absorbance values for each pixel and each wavenumber.

### Data pre-processing

Data pre-processing was applied to improve the robustness and the accuracy of the fusion model. The data pre-processing procedure detected and removed outlier spectra defined using a quality or “outlier” test that has an upper and lower threshold value of the Amide I band absorption intensity; spectra with absorbance intensity outside the range 0.1–2.0 were removed from the data cube. The noise was reduced by using principal component analysis noise reduction with 15 principal components, and then a rubber-band baseline correction was applied.^26^ Each spectrum was corrected for Mie scattering with one iteration;^27^ truncated to the fingerprint region (1800–1000 cm^−1^); vector normalised and then the paraffin region (1495–1350 cm^−1^) was excluded from the data cube.

### Image alignment

To apply the image fusion methodology, the ROIs must be identical in both optical and IR images. Any misalignment between images was corrected by using a geometric transformation within image editing software (Adobe Photoshop 2021 v22). The IR image at 1650 cm^−1^ provides the highest contrast in the IR image data cube and is referred to as the fixed image; the stained H&E image is referred to as the moving image. While using sequential sections minimises differences between the ROIs in both images, the staining methodology may introduce some distortion to the H&E images as the rehydration step can cause some expansion in the sections.

### Fusion model

**Fig. 1:**
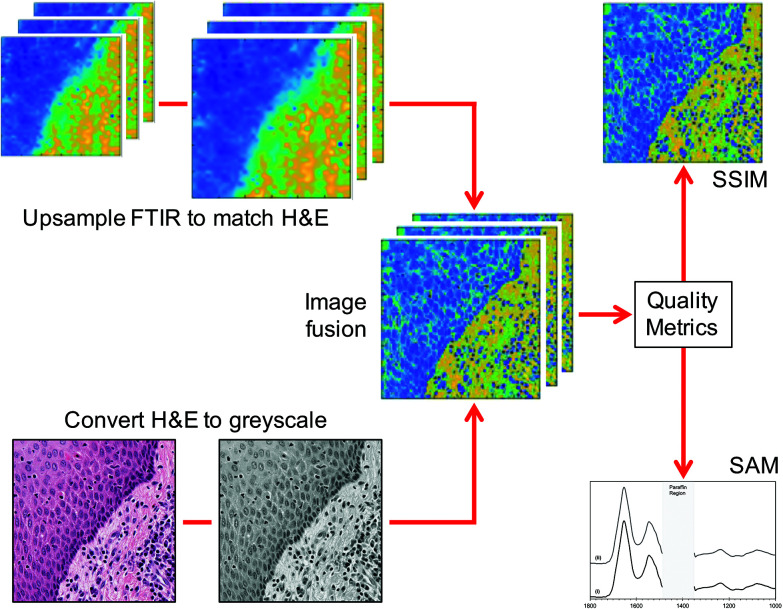
Flow diagram of the fusion process. After fusion the quality metrics are calculated for the images (structural similarity index measure, SSIM) and the spectra (spectral angle mapper, SAM).

The FTIR images were upsampled to the same scale as the H&E images using bicubic interpolation. The H&E images were converted to greyscale and then standardised (scaled) by subtracting the mean and dividing by the standard deviation. A fusion model^28^ that is based on linear regression with weighted slopes and intercepts was used to merge the images pixel by pixel. Any spectral distortion that might have resulted from fusing the images was mitigated by equalising the fused image to the FTIR image. The fused image underwent a quality test to eliminate spectra with absorbance intensities less than 0.1. When the fusion was complete quality metrics were applied to both the images and the spectra to ensure that neither displayed any significant distortion. The fusion workflow is shown in Fig. 1.

### Quality metrics

Spatial and spectral quality metrics were used to examine the results of the image fusion. Image quality metrics were implemented using a full-reference structural similarity index measure (SSIM)^29^ – part of the Python *scikit-image* image processing library^30^ – with H&E images used as a reference image (ground truth). SSIM is widely used in super-resolution imaging and was considered a good indication of how well the fused images correlate spatially with the H&E. A SSIM score of unity indicates that both images are identical; a lower value means less structural similarity between the images.

The spectral distortion was quantified using a spectral angle mapper (SAM) that measures spectral similarity, pixel-by-pixel, based on calculating the angular distortion between spectra treated as vectors in multidimensional space,^31^ and was implemented using the MATLAB *Image Processing Toolbox*.^32^ Identical spectra have a SAM score of zero, and higher values mean that there is some distortion in the spectral data. In this study, the FTIR images were used as a reference image to analyse any spectral distortion in the fused images.

## Results

**Fig. 2:**
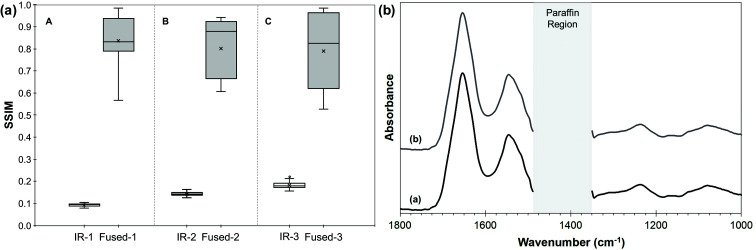
(a) The SSIM quality metric scores for the three different tissues A, B and C, before and after fusion. In each case, the reference image is the H&E. (b) Average spectra (i) before and (ii) after fusion for tissue A.

After FTIR data pre-processing, images were obtained at 174 wavenumbers in the range 1800–999 cm^−1^ (omitting the paraffin fingerprint region) resulting in a data spacing of ∼4 cm^−1^. Fig. 2 shows the quality metrics for the fused images and the spectra. The SSIM scores for the fused images from the three tissues studied [Fig. 2(a)] range from ∼0.6 to ∼1.0. The values are expected to be less than unity as the contrast components between the optical H&E image and the fused images will not be identical due to the importance of maintaining chemical information from the FTIR image. Nevertheless, the scores for the fused images are significantly higher than those of the FTIR images, which range from ∼0.1 to ∼0.2. The average spectra for tissue A before and after image fusion [Fig. 2(b)] are almost indistinguishable. Quantifying the degree of distortion that resulted from the image fusion, SAM values in the range 0.02–0.03 indicate that the distortion is minimal. For comparison, when comparing spectra from different tissues (that appear superficially similar) the SAM values were found to be 0.2–0.9.

**Fig. 3:**
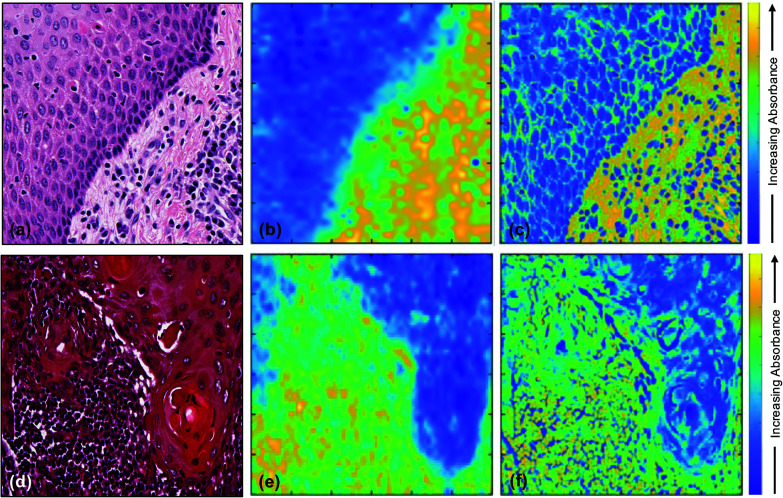
(a) H&E stained image of non-tumoural oral epithelium and underlying stroma with inflammatory/immune cells (tissue A); (b) corresponding FTIR image at 1242 cm^−1^; (c) fused image of (a) and (b); (d) H&E stained image of cervical lymph node metastasis of OSCC (tissue B); (e) corresponding FTIR image at 1242 cm^−1^; and (f) fused image of (d) and (e).

Fig. 3 shows the results of the image fusion at one wavenumber to demonstrate how effectively the spatial resolution of the H&E is combined with the spectral (chemical) information of the FTIR images. The increased spatial resolution in the fused images [Fig. 3(c) and (f)] compared to the FTIR images [Fig. 3(b) and (e)] reveals spatially-resolved differences in chemistry that were not apparent in the original FTIR.

The top left corner of the H&E image [Fig. 3(a)] shows non-tumoural oral epithelium which is expected to contain DNA and RNA but no collagen, while the bottom right corner shows connective tissue and inflammatory cells which are expected to contain collagen, DNA and RNA.

**Fig. 4:**
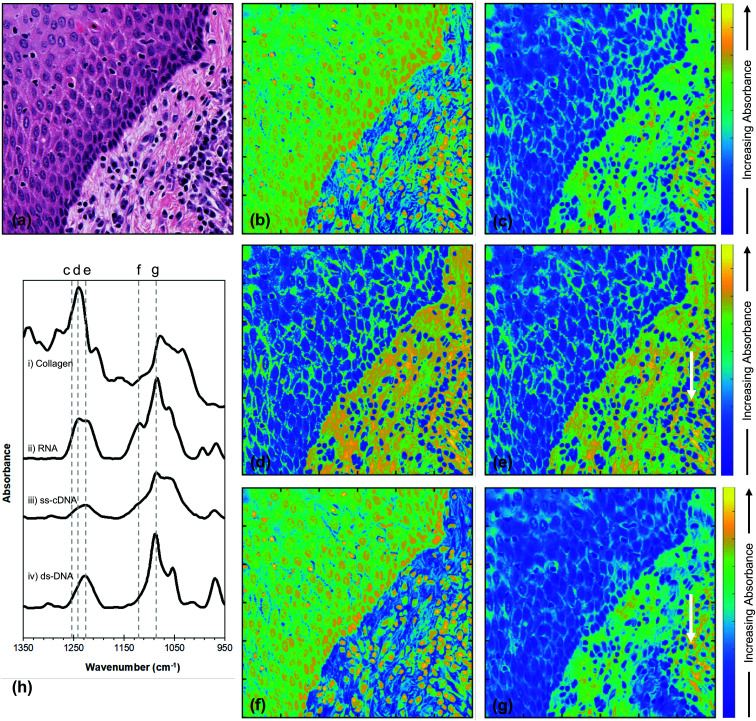
(a) H&E section of non-tumoural oral epithelium (top left) and underlying stroma (bottom right). The FTIR images chosen for image fusion were at wavenumbers of (b) 1717 cm^−1^, (c) 1252 cm^−1^, (d) 1242 cm^−1^, (e) 1225 cm^−1^, (f) 1119 cm^−1^ and (g) 1084 cm^−1^. (h) FTIR spectra of (i) type I collagen adapted from ref. 34, (ii) RNA, (iii) ss-cDNA and (iv) ds-DNA adapted from ref. 33. The dashed grey lines indicate the wavenumbers used for the fusion images. The white arrows in (e) and (g) indicate an inflammatory cell.

To gain insight into the spatial distribution of specific molecules which make important contributions to the chemistry of tissues (in this case, nucleic acids and collagen) fused images are presented in Fig. 4 at wavenumbers characteristic of these chemical moieties, together with the FTIR spectra of these moieties taken from the literature [Fig. 4(h)].^33,34^ The wavenumbers selected were 1717 cm^−1^; 1252 cm^−1^; 1242 cm^−1^; 1225 cm^−1^; 1119 cm^−1^ and 1084 cm^−1^.

## Discussion

The effectiveness of fusing FTIR images with those of the H&E stain is demonstrated by the high level of detail that can be resolved in the fused images of Fig. 4 and is confirmed by the values of the SSIM scores for these images being much higher than those of the original FTIR images. In addition, the low SAM values show that the process of fusing the FTIR images has produced very little spectral distortion, indicating that the integrity of the chemical information contained in the FTIR spectra has not been compromised by the image fusion. This combination of quality metrics provides justification for interpretation of the fused images at wavenumbers that are characteristic of specific molecules of relevance to the chemistry of tissues and at a spatial resolution usually associated with optical microscopy. In order to assess the extent to which the image fusion process provides insight into the chemical structure of tissue, the results obtained at a number of specific wavenumbers (Fig. 4) are compared with the image obtained from the H&E stain [Fig. 4(a)]. The top left corner of the H&E image shows non-tumoural oral epithelium which is expected to contain DNA and RNA, but no collagen, while the bottom right corner shows connective tissue stroma with inflammatory cells which are expected to contain collagen, DNA and RNA. The clear distinction between these two regions as appreciated by the H&E stain is reproduced in all the fused images shown in Fig. 4, although the overall difference in contrast between the two regions is dependent on the wavenumber at which the individual fused image is obtained. These wavenumbers (1717 cm^−1^; 1252 cm-1; 1242 cm^−1^; 1225 cm^−1^; 1119 cm^−1^; and 1084 cm^−1^) were chosen for their potential to reveal insight into the chemical structure of the tissue on the basis of the FTIR spectra of nucleic acids^33^ and collagen^34,35^ [Fig. 4(h)].

It is important to note that, while each fused image shows the variation in total absorbance within the tissue at a particular wavenumber, the variation in the absolute intensities of absorbance between different wavenumbers is not captured by the image fusion process. Similarly, the FTIR spectra of nucleic acids and collagen shown in Fig. 4(h) only show the variation in intensity with wavenumber within each moiety and not the relative intensities of nucleic acid and collagen spectra. However, within these restrictions, the variation in the distribution of collagen and nucleic acids within each fused image corresponds very well with that expected from Fig. 4(a), based upon our current knowledge of mucosal biology.

The fused images at wavenumbers 1717 cm^−1^ and 1119 cm^−1^, which are almost identical, show increased absorbance of these wavenumbers (yellow colour) in the cell nuclei in both epithelium and stroma [Fig. 4(b) and (f)], leading to a relatively higher overall absorbance of these wavenumbers in the epithelium compartment compared to the stroma. In contrast, fused images demonstrate relatively low absorbance of wavenumbers 1252 cm^−1^, 1242 cm^−1^, 1225 cm^−1^ and 1084 cm^−1^ by the cell nuclei in all types of tissue [blue colour; Fig. 4(c), (d), (e), and (g)], while the extracellular matrix in stroma absorbs these wavenumbers more strongly than the epithelium. The absorbance related to a cytoplasmic location varies and results in perinuclear green rims in both epithelium and stroma, whereas nuclei of occasional inflammatory cells (Fig. 4(e) and (g), arrow) seem rimmed by a yellow area. These variations reflect the difference between high and moderate absorption of the named wavenumber.

The variations in contrast within each image and between images can be explained by considering the FTIR spectra in Fig. 4(h). The 1119 cm^−1^ wavenumber, which arises from the ribose moiety and is highlighted by the dashed line f in Fig. 4(h), is expected to be strongly absorbed by RNA, but only weakly absorbed by DNA or collagen. The corresponding fusion image shows more absorption by the cell nuclei than cytoplasm or the extracellular matrix, with stronger absorption by the less differentiated (progenitor) epithelial compartment (cells of the basal, epithelial layer adjacent to the stroma) compared to the more differentiated (maturation) epithelial compartment (cells of the upper epithelial layers) [Fig. 4(f)]. Given that this wavenumber is not strongly absorbed by DNA and in accordance with current understanding of differentiation events in oral epithelium, we might hypothesise that cells in the maturation compartment are less transcriptionally active than those in the progenitor compartment or inflammatory/immune cells. The variation in absorbance in the fused image obtained at the 1717 cm^−1^ wavenumber [Fig. 4(b)] accords with this interpretation. At this wavenumber, collagen yields only a very weak spectral peak or no peak at all,^34,35^ whereas there is a significant contribution from DNA in this region of the IR spectrum.^2,36^ The high absorbance signal in the fusion image at 1717 cm^−1^ is thus expected to derive predominantly from the DNA, and is indeed preferentially associated with the nuclei of all types of cell, though more transcriptionally active in those of the basal epithelial layer and inflammatory cells.

The contrast within the fused image obtained at the wavenumber 1242 cm^−1^ [Fig. 4(d)] can be similarly interpreted in terms of the FTIR spectra [Fig. 4(h)] and the structure/organisation of the tissues obtained from the H&E stain [Fig. 4(a)]. The nucleic acids show strong contributions at this wavenumber [dashed line d Fig. 4(h)] and this is the strongest peak of the collagen spectrum. Since nucleic acids and collagen are both expected to contribute to absorbance at this wavenumber, the observation that the absorbance is strongest (yellow) in the stroma, particularly in the extracellular matrix in the bottom right of Fig. 4(d), indicates that collagen dominates the image contrast. This also explains the weaker absorbance in the nuclei of the epithelial and inflammatory cells, which do not contain collagen.

Inspection of the contrast within the fused images indicates that the difference in absorption between the bottom left and top right decreases in the sequence 1242 cm^−1^, 1225 cm^−1^, 1252 cm^−1^, 1084 cm^−1^ (compare Fig. 4(d), (e), (c) and (g), respectively). This sequence is expected from the variations in the FTIR spectra of collagen and nucleic acids as shown by the changes in relative intensities indicated by the dashed lines d, e, c and g in Fig. 4(h). It is reasonable to suppose that, since the peak in the collagen spectrum occurs at 1242 cm^−1^, then the fused image at this wavenumber [Fig. 4(d)] illustrates the maximum contribution from collagen. This collagen peak is narrower than the nucleic acid peaks in this spectral region and, while the nucleic acids spectral absorbances at 1225 cm^−1^ are either unchanged or accentuated compared to those at 1242 cm^−1^, the collagen absorbance falls by about 50%. This explains the reduction in absorbance in the stromal compartment (bottom right) of Fig. 4(e) compared to Fig. 4(d). The spectral intensities at 1252 cm^−1^ [dashed line c in Fig. 4(h)] show a further and stronger reduction in the collagen peak accompanied by a fall in the spectra of the nucleic acids. These changes are consistent with a further reduction in the collagen contribution to the absorbance and account for the reduction in the difference in intensity between epithelium and stroma in Fig. 4(c) compared to Fig. 4(d) and (e). This difference in intensity is further reduced in the fused image obtained at 1084 cm^−1^ [Fig. 4(g)]. At this wavenumber the nucleic acids and collagen all show strong spectral features. However, while the collagen peak at 1084 cm^−1^ is weaker than the peak at 1242 cm^−1^ (Fig. 4(h), lines g and d), the nucleic acid peaks are all significantly stronger at 1084 cm^−1^ than they are at all four of the wavenumbers considered previously. These observations are consistent with the observed further reduction in the difference in intensity between the two regions of the tissue in the fused image of Fig. 4(g) compared with Fig. 4(d), (e) and (c).

In general, the FTIR spectra of biological molecules are complex, showing significant overlaps, and there will be contributions to the chemistry of the tissue analysed in Fig. 4 other than collagen, such as glycogen and proteins. Indeed, the FTIR spectra shown by Wang *et al.*^36^ indicate that there could be a weak contribution to absorbance at 1084 cm^−1^ [Fig. 4(g)] from collagen and a weak contribution from glycoproteins in the regions of the other nuclei acid peaks. These weak contributions, if present, would contribute to the intensity but do not negate our observations that the intensity distributions within the fused images shown in Fig. 4 are consistent with the expected contributions from nucleic acids and collagen.

The analysis described here has used only images obtained at individual wavenumbers. More insight may be obtained by multivariate analysis of the absorbances at different wavenumbers that are characteristic of specific biomolecules.

## Conclusions

We have shown that pixel-level full image integration, or fusion, can generate images which combine the spatial resolution of optical microscopy images of H&E stained tissue with the chemical information in FTIR images. This obviates the diffraction limit on the spatial resolution of the FTIR images and provides significant additional information on the chemical structure of the tissue. Image fusion results obtained on three test specimens with oral squamous cell carcinoma (OSCC) at a single wavenumber showed that the spatial resolution of H&E stained images could be transferred to FTIR images and that any distortion in the fused images was minimal.

FTIR spectroscopy has a long history of revealing biomolecular information but its application to the determination of the chemical structure of tissue has been impeded by diffraction limits on the spatial resolution of FTIR images and by the overlapping of the FTIR spectra of different chemical moieties. This work has shown that the diffraction limit can be overcome by image fusion. In addition, the demonstration that a consideration of the FTIR spectra of nucleic acids and collagen [Fig. 4(h)] can explain the changes in contrast within and between each image (Fig. 4) shows that the second limitation can also be overcome in an important region of the IR spectrum.

The ability to exploit the rich spectral information of FTIR at a spatial resolution beyond that achievable with FTIR microscopy has huge potential for molecular mapping of biomolecules at sub-micron resolutions. Clearly this image fusion technique is not limited to H&E staining and could be applied more widely to other histochemical techniques and special stains to obtain complementary information to that demonstrated here.

## Conflicts of interest

There are no conflicts to declare.

## Supplementary Material
